# Comprehensive structural and functional analyses of RAD50 nsSNPs: from prediction to impact assessment

**DOI:** 10.3389/fbinf.2025.1535482

**Published:** 2025-03-26

**Authors:** Samina Malik, Mirza Jawad Ul Hasnain, Gul Zaib, Haleema Saadia, Arif Malik, Ayesha Zahid

**Affiliations:** ^1^ University College of Medicine and Dentistry, The University of Lahore, IMBB, UOL, Lahore, Pakistan; ^2^ Department of Biological Sciences, Virtual University of Pakistan, Islamabad, Pakistan; ^3^ Center for Bioinformatics and Computational Biology, Shanghai Key Laboratory of Regulatory Biology, Institute of Biomedical Sciences and School of Life Sciences, East China Normal University, Shanghai, China; ^4^ School of Pain and Regenerative Medicine, The University of Lahore, IMBB, UOL, Lahore, Pakistan

**Keywords:** breast cancer, Rad50, MRE11A, docking, simulation

## Abstract

**Background:**

The RAD50 gene on chromosome 5q3.11 plays an important role in the MRN (Mre11–Rad50–Nbs1) complex. This complex orchestrates cellular responses to the DNA double-strand breaks (DSBs) through several pathways for genome stability. This study aims to investigate the functional impact of non-synonymous single-nucleotide polymorphisms (nsSNPs) in RAD50 (a breast cancer-associated gene) and focuses on their consequences on protein structure and interaction within the MRN complex.

**Methods:**

A total of 1,806 nsSNPs were retrieved and subjected to variant analysis using a set of computational tools and ConSurf. Pathogenicity and protein stability criteria were established based on specific tools. Highly conserved damaging nsSNPs were prioritized for the structural analysis. GOR-IV was used for secondary structure prediction, whereas AlphaFold, RoseTTAFold, and I-TASSER were used for protein structure prediction. The docking of RAD50–Mre11A complexes was performed using HADDOCK to assess the impact of nsSNPs on protein–protein interactions. Molecular dynamic simulation was performed to verify the role of mutants in molecular docking analysis.

**Results:**

A subset of pathogenic and disease-associated nsSNPs in the RAD50 gene altered the protein stability and interactions with the Mre11A protein. Substantial alterations in the interacting profiles of mutants (A73P, V117F, L518P, L1092R, N1144S, and A1209T) suggest potential implications for DNA repair mechanisms and genome stability.

**Conclusion:**

The study discloses the normative impact of RAD50 mutations on the pathophysiology of breast cancer. It can provide the basis to treat RAD50 mutation-deficient cells.

## 1 Introduction

The MRN (Mre11–Rad50–Nbs1) complex’s essential protein component, which diligently maintains DNA stability aboard the nourishing genome, has organizational ethics (McCarthy et al., 2022). The DNA double-strand break repair method relies upon this complex for carrying out the function of significant genome stability (Qiu and Jun 2021).

The MRN complex stimulates cell responses to DSBs through several nerve pathways, including DNA damage checkpoint activation, together with HR and NHEJ. The RAD50 protein belongs to a dynamic complex with Mre11 and Nbs1 (nibrin) in order to facilitate DNA repair function near the site of damage (Qiu and Jun 2021; Bian et al., 2019). The hRAD50 inherited sequence generates a 1,312-amino acid inside the human body (a 153-kDa RAD50 protein). ATP-regulated changes in the unlocked and close position of the RAD50 protein restrict MRE11’s ability to perform nucleolysis tasks (Deshpande et al., 2014). RAD50 molecules, along with the ATP boundary in the head of each RAD50 monomer, exhibit a solid closed conformation. The DNA-interacting sphere of these proteins unites to form a major double-stranded deoxyribonucleic acid adhesion groove (Cassani et al., 2019). Organisms with a human RAD50 biological mutant show increased radiation sensitivity and decreased ability to repair double-strand DNA interruption while preserving chromosomal uncertainty. Cancer development may be influenced by the RAD50 gene mutant, causing a functional disorder (Ragamin et al., 2020).

Cancer susceptibility increases with genomic volatility diseases, which combine immunologic impairment with radiation sensitivity, chromosome instability, and ancestral loss. The MRE11 gene closure causes an ataxia-telangiectasia-like condition similar to the observation in Alblihy et al. (2021). The NBS1 mutant produces three features that include a progressive development of microcephaly, a low-to-moderate decreased stature, and an abnormal facial framework. The latter causes a brow slope on the upper jaw and an increase in ear size, called Nijmegen breakage syndrome (NBS). Laboratory studies have shown that RAD50 does not lead to diseases similar to NBS (Bian et al., 2019). Scientists must first understand how the RAD50 mutant alters cellular processes and the disease growth nerve pathway to develop targeted therapy for an ancestral disease (Chisada et al., 2023).

Scholars investigating the concentration of SNPs over DNA have been sequencing nucleotide location changes to investigate their functional implications (Chaudhary et al., 2015). SNPs are a widespread hereditary variation in the human genome, with the frequency increasing with every population group. NsSNPs are the most important type of SNPs as they modify the sequence of the encoded protein (Hassan et al., 2019). Protein structure, as well as nerve pathway molecule communication, may be altered by a discrepancy in its basic organization, which occurs consequent to DNA sequence changes (Stollar and Smith, 2020).

We studied variations in Mre11A string A interconnectivity within the MRN multicomplex. In the *in silico* strategy, variant analysis was performed to assess pathogenicity, protein uncertainty, and diseases associations using a broad range of instruments such as CADD (Rentzsch et al., 2019), PolyPhen (Adzhubei et al., 2013), SIFT (Sim et al., 2012 CE), PANTHER (Mi et al., 2021), ESM1b (Livesey and Marsh, 2023), AlphaMissense (Cheng et al., 2023), EVE (Frazer et al., 2021), SNPs&GO (Capriotti et al., 2013), PhD-SNP (Capriotti and Fariselli, 2nd ed., 1997), I-Mutant2.0 (Cheng et al., 2006), MUpro (Ashkenazy et al., 2016), and ConSurf (Ruff and Pappu, 2021). As part of the organizational examination, protein structure prediction and protein–protein docking were performed.

## 2 Materials and methods

### 2.1 Data retrieval

To date, 35,054 SNPs of the RAD50 gene have been reported. Among them, 1,896 SNPs were reported as non-synonymous. Genome variant information, including RS_IDs, chromosome number, variant positions, and variants, was retrieved from the NCBI database. Protein sequence-based information was retrieved from UniProtKB. A total of 12 computational tools, three of which were AI-based, were used to find the deleterious and pathogenic effects of the nsSNPs, as well as their impact on protein stability and disease association.

### 2.2 Protein functional analysis

#### 2.2.1 Deleterious and pathogenic effects of nsSNPs

CADD, PolyPhen, SIFT, PANTHER, ESM1b, AlphaMissense, and EVE (score, ASM, and clinical significance) were used for functional analysis, which included the deleterious and pathogenic effects of nsSNPs. All tools were run on default values, and the criteria of the selection were based on certain parameters. For CADD, we chose nsSNPs having a Phred score greater than 20 (variants predicted to be among the top 1% most deleterious substitutions in the human genome). The nsSNPs with PolyPhen score >0.8, SIFT deleterious score <0.1, and ESMb1 value < −7.5 were selected as most pathogenic. PANTHER and AlphaMissense only identified likely pathogenic and possibly damaging nsSNPs, with cut-off values greater than 0.5. The EVE tool classified nsSNPs with a score greater than 0.7 as pathogenic, with the most confident pathogenic score indicating clinically significant pathogenicity. Evolutionary conservation patterns of amino acid residues in the RAD50 protein sequence were observed by use of the ConSurf tool. Highly conserved variants in the protein sequence with scores greater than 7 were chosen. The nsSNPs verified by at least seven out of eight tools based on the prescribed cut-off criteria proceeded to the next step.

#### 2.2.2 Prediction of protein instability and disease association of nsSNPs

PhD-SNP and SNPs&GO were used to assess the neutral and pathogenic effects of the highly deleterious nsSNPs. MUpro and I-Mutant2.0 tools were used to assess the protein stability affected by the pathogenic variants. Those nsSNPs that were verified as disease-causing and associated with protein instability by four tools were selected.

Overall, the variants qualifying at least 11 out of 12 tools were used to investigate the structural impact of damaging nsSNPs in the RAD50 protein.

### 2.3 Structural analysis of damaging nsSNPs in the RAD50 protein

To find the impact of nsSNPs at the amino acid level, GOR-IV, a web server (https://npsa-prabi.ibcp.fr/NPSA/npsa_gor4.html), was used for the secondary structure prediction of native and mutant RAD50 protein sequences. Previously, there was no complete protein structure of the RAD50 protein available in the Protein Data Bank (PDB) and AlphaFold. Thus, the full-length amino acid sequence of the RAD50 protein was predicted through three AI-based protein structure tools, namely, AlphaFold (Du et al., 2021), RoseTTAFold (Yang et al., 2015), and I-TASSER (Van et al., 2011). For long sequences, AlphaFold and RoseTTAFold use deep learning protein prediction models through multiple sequence alignment and transformer neural network with template-free folding prediction, respectively. I-TASSER focuses on threading, *ab initio* modeling, and structure refinement methods. Global quality was assessed through the Protein Structure Validation Suite (Z-score) and Expasy QMEAN score. Local residual quality was assessed through RAMPAGE (stereochemical quality of proteins by the Ramachandran plot) to obtain the best protein models of RAD50 (native and mutant). Models were then normalized through 3Drefine. FoldX (De et al., 2010) was used for mutagenesis. Mre11A chain A binding with Rad50 domains in the MRN complex was downloaded from the Protein Data Bank, and the same Mre11 domain was docked with our native and mutant models to observe the residual alterations in the docked complexes. Docking was performed through HADDOCK (Abraham et al., 2015), an online tool that employs an integrative modeling approach to extract information from biochemical data, mutagenesis studies, and prediction models. HADDOCK is capable of operating both rigid-body and flexible docking, allowing leverage for the conformational changes in the interacting models during the docking process.

### 2.4 Molecular dynamic simulation

Molecular dynamic simulations of RAD50 wild-type and mutant protein structures were performed using the default parameters of the GROMACS 5.1.4 (Kuhlman and Bradley, 2019) package on the Linux workstation with 32 GB RAM and Intel Core i9-11950H Processor. Systems were kept as default in a rectangular box of 10 Å marginal radius. Conformational changes were observed for a time scale of 100 ns with 500000 n steps, and the protein stability was observed after every 10 steps 2fs. Comparative structural analysis was carried out using grms, grmsf, ggyrate, and gsasa tools. Graphs were plotted using Grace GUI toolkit 5.1.22.

## 3 Results

**FIGURE 1:**
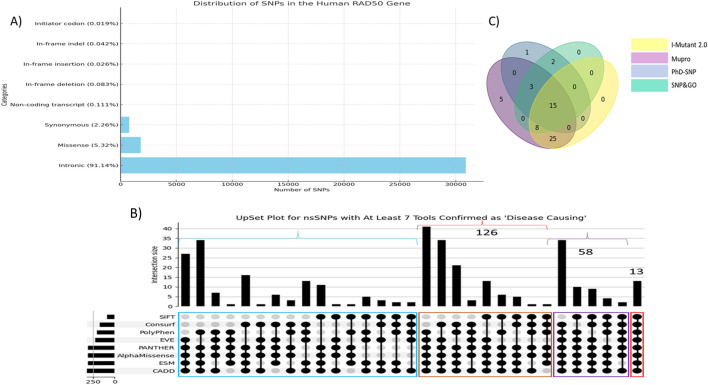
**(A)** Distribution of single-nucleotide polymorphisms in the human RAD50 gene. **(B)** UpSet plot of top 350 nsSNPs. Red color highlighted nsSNPs verified by all eight tools. Purple color showed nsSNPs verified by at least seven colors, whereas brown and blue colors highlight the number of nsSNPs verified by at least six and five tools, respectively. **(C)** Venn diagram showed 15 out of 71 nsSNPs found as disease-associated and linked to protein instability verified by I-Mutant2.0, MUpro, PhD-SNP, and SNPs&GO.

NCBI showed 35,054 human RAD50 gene SNPs. Upon filtration, most of the SNPs were observed in the intronic region (30,901, 91.14%), whereas the second- and third-most SNPs are missense (1,804, 5.32%) and synonymous (762, 2.26%), respectively. Other variants were very few, including non-coding transcript variants (0.111%), in-frame deletion (0.083%), in-frame insertion (0.026%), in-frame indel (0.042%), and initiator codon variants (0.019%). Figure 1A shows a graphical representation of the distribution of human RAD50 SNPs.

### 3.1 Deleterious and pathogenic effects of nsSNPs

The pathogenicity and deleterious impacts of all nsSNPs were found using eight tools as described in the section on methodology. CADD reported 1,025 variants having a Phred score greater than 20, whereas PolyPhen (score>0.8), SIFT (score<0.1), and ESM1b (score<−7.5) reported 500, 331, and 509 variants, respectively. The clinically significant variants predicted by AlphaMissense, PANTHER, EVE_classes_75_pct_retained_ASM, and EVE were 431, 395, 474, and 383, respectively. ConSurf reported 554 variants in highly conserved regions in the protein sequence. A total of 13 variants were found as pathogenic predicted by all eight tools, whereas 58 variants were predicted as pathogenic from at least 7 out of 8 tools. Figure 1B depicts the UpSet plot of most deleterious nsSNPs verified by maximum tools.

### 3.2 Prediction of protein instability and disease association of nsSNPs

These 71 nsSNPs were then subjected to analysis by SNPs&GO, PhD-SNP, I-Mutant 2.0, and MUpro to find disease effect and protein instability. Figure 1C shows a Venn diagram of 15 out of 71 nsSNPs predicted as disease-causing and associated with protein instability verified by 4 tools. Subsequently, these 15 variants showing disease-associated impact and decreasing protein stability were subjected to structural analysis (Table 1).

**TABLE 1:** Predicting functional impact, disease association, protein stability, and sequence conservation of nsSNPs in the RAD50 gene. “C,” “D,” “DCR,” “DLT,” “L_P,” “P,” “Prob_D,” “Poss_D,” and “T” stands for conflicting interpretations of pathogenicity, disease, decrease, deleterious, likely pathogenic, pathogenic, probably damaging, possibly damaging, and tolerated.

dbSNP	Gene variant	AA variant	Functional consequences of nsSNPs								Disease-associated nsSNPs			
			CADD Phred score >20	PolyPhen score>0.8	SIFT score<0.1	ESM1b score	AlphaMissense	PANTHER	EVE 75_pct retained Clinical significance	ConSurf conservation score	MUpro protein stability	I-Mutant2.0 protein stability	PhD-SNP Disease association	SNPs&GO Disease association
rs2149866892	T/C	S1202T	31	Prob_D	DLT	−15.466	L_P	Poss_D	P (L_P)	9	DCR	DCR	D	D
rs1581023680	T/C	L1215P	32	Prob_D	DLT	−17.184	L_P	Poss_D	P (L_P)	9	DCR	DCR	D	D
rs587782311	A/G	L518P	20.7	Poss_D	DLT	−13.548	L_P	Poss_D	P (L_P)	9	DCR	DCR	D	D
rs1554100957	G/A	M1197I	29.7	Prob_D	DLT	−11.697	L_P	Poss_D	P (L_P)	8	DCR	DCR	D	D
rs762302203	T/C	Y1155D	27.8	Prob_D	DLT	−15.056	L_P	Poss_D	P (L_P)	9	DCR	DCR	D	D
rs587780156	G/A	R1156L	32	Prob_D	DLT	−10.576	L_P	Poss_D	P (C)	9	DCR	DCR	D	D
rs376109008	A/G	N1144S	26.5	Prob_D	DLT	−11.451	L_P	Poss_D	P (L_P)	9	DCR	DCR	D	D
rs751641472	T/C	M1197R	27.4	Prob_D	DLT	−16.999	L_P	Poss_D	P (L_P)	8	DCR	DCR	D	D
rs371122101	G/A	A73P	27.8	Poss_D	DLT	−11.977	L_P	Poss_D	P (L_P)	9	DCR	DCR	D	D
rs876659810	T/C	L1092R	27.3	Prob_D	DLT	−12.802	L_P	Poss_D	P (L_P)	8	DCR	DCR	D	D
rs2149841115	G/T	A1209T	28.7	Prob_D	T	−6.476	L_P	Poss_D	P (L_P)	9	DCR	DCR	D	D
rs587782295	A/G	V117F	22.1	Poss_D	DLT	−8.022	L_P	Poss_D	P (L_P)	9	DCR	DCR	D	D
rs397507179	G/A	D1170H	26.6	Poss_D	DLT	−11.093	L_P	Poss_D	P (L_P)	9	DCR	DCR	D	D
rs1554101183	G/A	G1226S	32	Poss_D	DLT	−10.275	L_P	Poss_D	P (L_P)	9	DCR	DCR	D	D
rs786201788	G/A	C133F	27.5	Poss_D	DLT	−10.188	L_P	Poss_D	P (L_P)	8	DCR	DCR	D	D

### 3.3 Structural analysis of damaging nsSNPs in the RAD50 protein

#### 3.3.1 Secondary structure analysis

**FIGURE 2:**
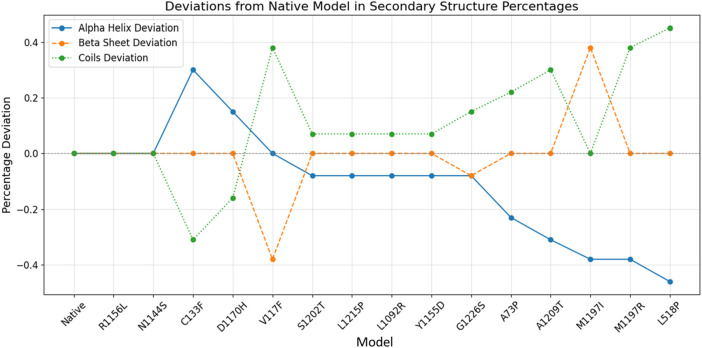
Secondary structure prediction of native and mutant RAD50 proteins. The blue line indicates the alpha helix deviation across the models, whereas brown and green lines indicate the deviation of beta sheets and coils across the models, respectively.

The GOR-IV tool showed that the secondary structures R1156L and N1144S are the same as in the native model. All three models have the same number of residues involved in the alpha helices, beta sheets, and coils. Although there is a shifting of residues between alpha helices and coils, no beta sheet residual change was found in the C133F, D1170H, S1202T, L1215P, L1092R, Y1155D, A73P, A1209T, M1197R, and L518P models (Table 2). The V117F model showed residual shifting between the beta sheet and coils. Models G1226S and M1197I showed residual shifting in all three secondary structural elements. Figure 2 depicts that models R1156L and N1144S had same number of alpha helices, beta sheets and coils. Ten models, namely, C133F, D1170, S1202T, L1215P, L1092R, Y1155D, A73P, A1209T, M1197R, and L518P, had shifting of amino acid residues between alpha helices and coils, keeping the number of residues in beta sheets the same. Among these 10 models, only C133F and D1170H showed the shifting of residues from coils to alpha helices, whereas other 8 models showed a shift of residues toward the coils. The model V117F showed a shift of residues from beta-sheets to coils, keeping the alpha content unchanged, whereas M1197I showed a shift of residues from alpha helices to beta sheets, keeping the coil content unchanged. Model G1226S on the other hand had showed a shift of residues among all three secondary structures.

**TABLE 2:** Secondary structure prediction of native and mutant RAD50 proteins.

Model	Alpha helix		Beta sheet		Coil	
	Residue	Percentage	Residue	Percentage	Residue	Percentage
Native	929	70.81	96	7.32	287	21.88
R1156L	929	70.81	96	7.32	287	21.88
N1144S	929	70.81	96	7.32	287	21.88
C133F	933	71.11	96	7.32	283	21.57
D1170H	931	70.96	96	7.32	285	21.72
V117F	929	70.81	91	6.94	292	22.26
S1202T	928	70.73	96	7.32	288	21.95
L1215P	928	70.73	96	7.32	288	21.95
L1092R	928	70.73	96	7.32	288	21.95
Y1155D	928	70.73	96	7.32	288	21.95
G1226S	928	70.73	95	7.24	289	22.03
A73P	926	70.58	96	7.32	290	22.1
A1209T	925	70.5	96	7.32	291	22.18
M1197I	924	70.43	101	7.7	287	21.88
M1197R	924	70.43	96	7.32	292	22.26
L518P	923	70.35	96	7.32	293	22.33

Alpha helices showed an overall deviation range of −0.44% to +0.31%, whereas beta sheets and coils showed an overall deviation range of −0.39% to +0.38% and −0.31% to +0.44%. Studies proved that changes in beta sheets are more significant in terms of protein misfolding (Moreno-Gonzalez and Soto, 2011), and we also observed that the structural variability of beta sheets is more pronounced compared to alpha helices and coils, making it the most affected secondary structure in some models. Overall, seven mutants (A73P, V117F, C133F, L518P, M1197I, M1197R, and A1209T) had more residual variation, with a percentage deviation greater than 0.2%, whereas six mutants (D1170H, S1202T, L1215P, L1092R, Y1155D, and G1226S) had less residual fluctuation, with a percentage deviation of less than 0.2%.

#### 3.3.2 Protein structure prediction and docking analysis

**FIGURE 3:**
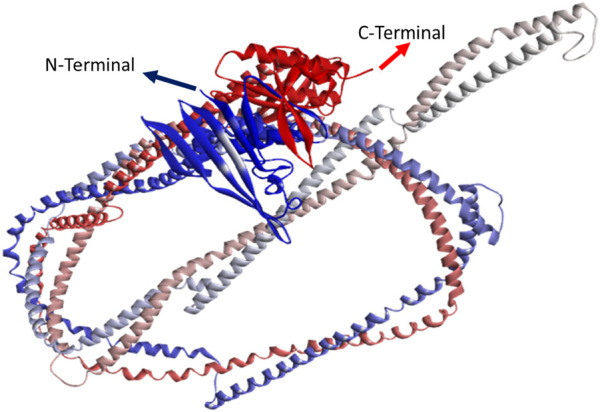
3D structure predicted by AlphaFold and RoseTTAFold. Blue color indicates N-terminal, whereas red color indicates C-terminal.

As there has previously been no experimental protein structure reported, the 3D structures of the RAD50 protein native model were downloaded through AlphaFold (AF-Q92878-F1) and predicted by RoseTTAFold and I-TASSER. The protein sequence retrieved from UniProtKB having 1,312 amino acids was used for protein structure prediction. We used the AlphaFold predicted model (AF-Q92878-F1) and partial X-ray structure of the hRAD50 protein (PDBID: 5GOX) as template models to predict the model through I-TASSER. Table 3 depicts the comparison of structural quality metrics for protein models generated by different modeling tools. By considering all factors, including Ramachandran plot summary, Z-score, and RMS deviation for bond angles and bond lengths, the RoseTTAFold model appears to be slightly superior. It has slightly higher percentages of favored regions in both Ramachandran plot summaries compared to I-TASSER and identical percentages when compared to AlphaFold. Additionally, RoseTTAFold had a slightly lower Z-score than both I-TASSER and AlphaFold, indicating better model quality. The RMS deviations for bond angles and bond lengths are comparable among all models. Since AlphaFold and RoseTTAFold had almost the same RMS, we analyzed the RMSD (root mean square deviation) of three models to determine how structures deviate from the reference conformations. It is found that the RMSD of AlphaFold and RoseTTAFold is almost the same, indicating that both predicted models have the same structural conformations and hence can be used interchangeably. So, we used the protein model verified by both AlphaFold and RoseTTAFold tools (Figure 3). FoldX was used to create mutant models through mutagenesis.

**TABLE 3:** Comparison of structural quality metrics for protein models generated by different modeling tools.

Tool	Ramachandran plot summary from PROCHECK				Ramachandran plot summary from Richardson Lab’s MolProbity			Z-score			RMS deviation for bond angle	RMS deviation for bond length	RMSD deviation for bond length
	Most favoured region	Additionally allowed region	Generously allowed region	Disallowed region	Most favoured region	Allowed region	Disallowed region	Verify3D	ProsaII	MolProbity clashscore			
I-TASSER	92.80%	6.60%	0.30%	0.30%	96.40%	3.40%	0.30%	−4.01	0	−7.19	1.4°	0.009 Å	1.13 Å°
RoseTTAFold	95.40%	4.20%	0.30%	0.20%	97.90%	1.70%	0.40%	−4.82	0.33	0.86	2.2°	0.021 Å	0.70Å°
AlphaFold	95.40%	4.20%	0.30%	0.20%	97.90%	1.70%	0.40%	−4.82	0.33	1.53	2.2°	0.021 Å°	0.70 Å°

**FIGURE 4:**
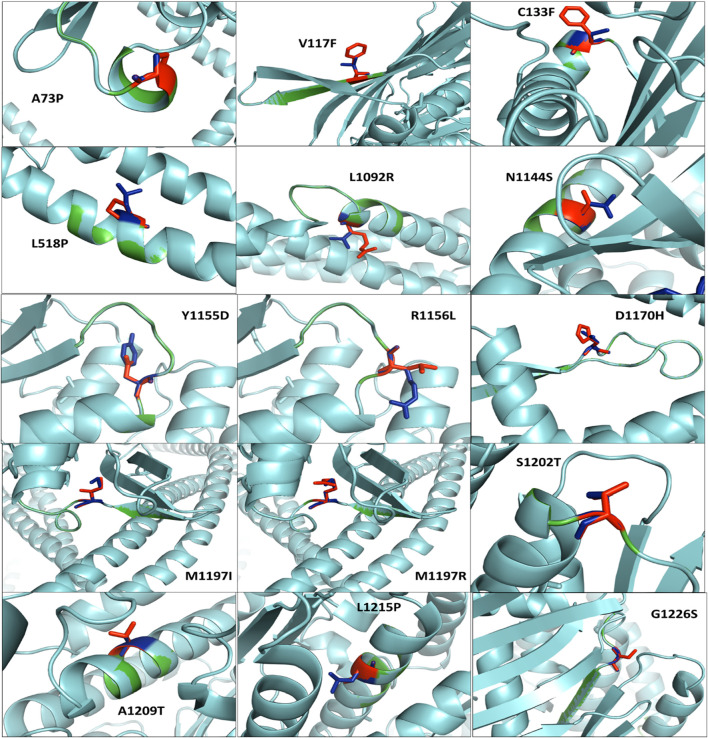
Superimposed residual of all mutant models (cyan color) with the native RAD50 protein model (green color). Native and mutant residues are colored blue and red, respectively.

Figure 4 shows the protein superimposition of all mutant models, with the native RAD50 protein model depicting those eight mutations, namely, A73P, V117F, C133F, L518P, L1092R, N1144S, A1209T, and L1215P, on the sheets. In addition, seven mutations, namely, R1156L, D1170H, M1197I, M1197R, S1202T, L1215P, and G1226S, were found in coil elements.

MRE11A chain A (PDB ID: 3T1I) was downloaded and docked with native and all 15 mutant RAD50 protein models after the refinement. Table 4 shows the overall interactions of RAD50 native and mutant models with the Mre11A chain in complexes.

**TABLE 4:** Interacting residues obtained from docking. Protein structures, as well as native and all mutant models of RAD50 protein with MRE11 chain A, including their binding residues and surrounding hydrophobic interactions. Receptors are RAD50 protein models, whereas ligands are MRE11 chain A.

Model	Receptor		Ligand		Binding free energy (ΔG) kcal/mol
	Hydrophobic interaction	Hydrophilic interaction	Hydrophobic interaction	Hydrophilic interaction	
Native + mre11	Gly200, Gln201, Val203, Lys204, Gln207, Met208, Leu210, Leu445, Glu448, Ile449, Lys453, Glu456, Glu893, Glu897, Met1111, Asn1118, Lys1126, Ser1176, and Asp1177	Arg196, Lys211, Lys452, Lys1119, and Asp1122	Phe354, Leu356, Lys357, Ile359, Ile361, Gly363, Glu364, Phe370, Tyr372, Ile374, Thr375, and Leu376	Asp360, Asp369, Lys375, Ile377, and Glu379	−29.4
A73P + mre11	Lys20, Glu75, Gln97, and Arg98	Glu212, Glu247, Arg248, Tyr249, and Phe265	GLu18, Tyr51, Ile52, Cys53, Gly55, Asp56, Phe57, Phe66, Asp77, Arg79, GLn81, Ala95, and Val96	Ala182, Agr183, Arg210, Tyr211, Ser214, Gly217, Pro219, Gly250, Val251, Asn252, Lys262, Pro263, and ARg264	−36.7
V117F + mre11	Lys6, Ile24,Gly39,Lys71, and Glu75	V117F Glu181, Gly184, Asp241, Tyr249, and Lys279	Arg13, Ser14, Phe15, Gly16, and Ile17 Ile25, Thr26, Phe28, Pro30, and Ile33 Val35, Pro37, Asn38, Ala40, and Pro70 Val72, Ala73, and Gln74	Glu178, Val179, Ala182, Arg183, Leu192, Arg210, Tyr211, Glu212, Thr,213, Ser214, Tyr215, Glu239, Trp240, Lys244, Lys246, Agr248, Gly250, Val251, Asn252, Gly280, Ser281, and Glu284	−35.9
C133F + mre11	Gly200, Gln201, Val203, Lys204, Gln207, Met208, Leu210, Leu445, Glu448, Ile449, Lys453, Glu456, Glu893, Glu897, Met1111, Asn1118, Lys1126, Ser1176, and Asp1177	Arg196, Lys211, Lys452, Lys1119, and Asp1122	Phe354, Leu356, Lys357, Ile359, Ile361, Gly363, Glu364, Phe370, Tyr372, Ile374, Thr375, and Leu376	Asp360, Asp369, Lys375, Ile377, and Glu379	−29.3
L518P + mre11	Val203, Lys204, Gln207, Leu210, Lys214, Glu393, Glu397, Leu445, Glu448, Ile449, Lys452, Lys453, Glu456, Met1111, Glu1115, Asn1118, Lys1126, Ser1176, and Asp1177	Arg196, Gly200, Lys211, Lys1119, and Asp1122	Phe354, Leu356, Lys357, Ile359, Ile361, Gly363, Glu364, Phe370, Tyr372, Ile374, Thr375, Leu376, and Leu378	Ile359, Asp360, Asp369, Tyr372, Lys375, and Glu379	−31.7
L1092R + mre11	Val203, Lys204, Gln207, Leu210, Lys214, Glu393, Glu397, Leu445, Glu448, Ile449, Lys452, Lys453, Glu456, Met1111, Glu1115, Asn1118, Lys1126, Ser1176, and Asp1177	Arg196, Gly200, Lys211, Lys1119, and Asp1122	Phe354, Leu356, Lys357, Ile359, Ile361, Gly363, Glu364, Phe370, Tyr372, Ile374, Thr375, Leu376, and Leu378	Ile359, Asp360, Asp369, Tyr372, Lys375, and Glu379	−31.6
N1144S + mre11	Val203, Lys204, Gln207, Leu210, Lys214, Glu393, Glu397, Leu445, Glu448, Ile449, Lys452, Lys453, Glu456, Met1111, Glu1115, Asn1118, Lys1126, Ser1176, and Asp1177	Arg196, Gly200, Lys211, Lys1119, and Asp1122	Phe354, Leu356, Lys357, Ile359, Ile361, Gly363, Glu364, Phe370, Tyr372, Ile374, Thr375, Leu376, and Leu378	Ile359, Asp360, Asp369, Tyr372, Lys375, and Glu379	−31.7
Y1155D + mre11	Gly200, Gln201, Val203, Lys204, Gln207, Met208, Leu210, Leu445, Glu448, Ile449, Lys453, Glu456, Glu893, Glu897, Met1111, Asn1118, Lys1126, Ser1176, and Asp1177	Arg196, Lys211, Lys452, Lys1119, and Asp1122	Phe354, Leu356, Lys357, Ile359, Ile361, Gly363, Glu364, Phe370, Tyr372, Ile374, Thr375, and Leu376	Asp360, Asp369, Lys375, Ile377, and Glu379	−29.3
R1156L + mre11	Gly200, Gln201, Val203, Lys204, Gln207, Met208, Leu210, Leu445, Glu448, Ile449, Lys453, Glu456, Glu893, Glu897, Met1111, Asn1118, Lys1126, Ser1176, and Asp1177	Arg196, Lys211, Lys452, Lys1119, and Asp1122	Phe354, Leu356, Lys357, Ile359, Ile361, Gly363, Glu364, Phe370, Tyr372, Ile374, Thr375, and Leu376	Asp360, Asp369, Lys375, Ile377, and Glu379	−29.3
D1170H + mre11	Gly200, Gln201, Val203, Lys204, Gln207, Met208, Leu210, Leu445, Glu448, Ile449, Lys453, Glu456, Glu893, Glu897, Met1111, Asn1118, Lys1126, Ser1176, and Asp1177	Arg196, Lys211, Lys452, Lys1119, and Asp1122	Phe354, Leu356, Lys357, Ile359, Ile361, Gly363, Glu364, Phe370, Tyr372, Ile374, Thr375, and Leu376	Asp360, Asp369, Lys375, Ile377, Glu379	−29.3
M1197I + mre11	Gly200, Gln201, Val203, Lys204, Gln207, Met208, Leu210, Leu445, Glu448, Ile449, Lys453, Glu456, Glu893, Glu897, Met1111, Asn1118, Lys1126, Ser1176, and Asp1177	Arg196, Lys211, Lys452, Lys1119, and Asp1122	Phe354, Leu356, Lys357, Ile359, Ile361, Gly363, Glu364, Phe370, Tyr372, Ile374, Thr375, and Leu376	Asp360, Asp369, Lys375, Ile377, and Glu379	−29.4
M1197R + mre11	Gly200, Gln201, Val203, Lys204, Gln207, Met208, Leu210, Leu445, Glu448, Ile449, Lys453, Glu456, Glu893, Glu897, Met1111, Asn1118, Lys1126, Ser1176, and Asp1177	Arg196, Lys211, Lys452, Lys1119, and Asp1122	Phe354, Leu356, Lys357, Ile359, Ile361, Gly363, Glu364, Phe370, Tyr372, Ile374, Thr375, and Leu376	Asp360, Asp369, Lys375, Ile377, and Glu379	−29.3
S1202T + mre11	Gly200, Gln201, Val203, Lys204, Gln207, Met208, Leu210, Leu445, Glu448, Ile449, Lys453, Glu456, Glu893, Glu897, Met1111, Asn1118, Lys1126, Ser1176, and Asp1177	Arg196, Lys211, Lys452, Lys1119, and Asp1122	Phe354, Leu356, Lys357, Ile359, Ile361, Gly363, Glu364, Phe370, Tyr372, Ile374, Thr375, and Leu376	Asp360, Asp369, Lys375, Ile377, and Glu379	−29.3
A1209T + mre11	Val203, Lys204, Gln207, Leu210, Lys214, Glu393, Glu397, Leu445, Glu448, Ile449, Lys452, Lys453, Glu456, Met1111, Glu1115, Asn1118, Lys1126, Ser1176, and Asp1177	Arg196, Gly200, Lys211, Lys1119, and Asp1122	Phe354, Leu356, Lys357, Ile359, Ile361, Gly363, Glu364, Phe370, Tyr372, Ile374, Thr375, Leu376, and Leu378	Ile359, Asp360, Asp369, Tyr372, Lys375, Glu379	−31–6
L1215P + mre11	Val203, Lys204, Gln207, Leu210, Lys214, Glu393, Glu397, Leu445, Glu448, Ile449, Lys452, Lys453, Glu456, Met1111, Glu1115, Asn1118, Lys1126, Ser1176, and Asp1177	Arg196, Gly200, Lys211, Lys1119, and Asp1122	Phe354, Leu356, Lys357, Ile359, Ile361, Gly363, Glu364, Phe370, Tyr372, Ile374, Thr375, Leu376, and Leu378	Ile359, Asp360, Asp369, Tyr372, Lys375, and Glu379	−31.7
G1226S + mre11	Gly200, Gln201, Val203, Lys204, Gln207, Met208, Leu210, Leu445, Glu448, Ile449, Lys453, Glu456, Glu893, Glu897, Met1111, Asn1118, Lys1126, Ser1176, and Asp1177	Arg196, Lys211, Lys452, Lys1119, and Asp1122	Phe354, Leu356, Lys357, Ile359, Ile361, Gly363, Glu364, Phe370, Tyr372, Ile374, Thr375, and Leu376	Asp360, Asp369, Lys375, Ile377, and Glu379	−29.3

The RAD50 protein contains specific regions and residues essential for binding to DNA and other proteins. There are several pockets in RAD50 proteins, including DNA-binding sites and protein–protein interaction sites. DNA-binding sites have two regions: the nucleotide binding domain, which binds with DNA through a strand–loop–helix motif with residues R94, K95, K99, K108, K109, K115, and S118, and the coiled-coil region, which contains lysine residues such as K175, K178, and K182 that contribute to DNA binding (Rojowska et al., 2014). The RAD50 protein interacts with other proteins either through MRE11 binding, in which the nucleotide-binding domains of RAD50 (K42, D63, K131, G144, K249, S250, and E802) and dimerization of MRE11 support the protein–protein interactions (Linh et al., 2017) and TRF2 interactions. In this study, the iDDR (the inhibitor of DNA damage response) motif of TRF2 (a human shelterin protein) interacts directly with the RAD50 protein (K125, R186, E308, D382, K450, R454, E456, and K464), leading to a convergent mechanism of binding with other proteins (Khayat et al., 2024).

Table 4 shows that none of the interacting residues in the docked complexes correspond to the known binding-site residues. It is observed that the native RAD50–Mre11A complex and eight C133F, Y1155D, R1156L, D1170H, M1197I, M1197R, S1202T, and G1226S mutant models docked with the Mre11A chain had the same pattern of hydrophobic and hydrophilic interactions. L1215P, A1209T, N1144S, L1092R, and L518P mutant models showed slightly altered residual interactions in RAD50–Mre11 complexes. On the other hand, V117F and A73P mutant–Mre11A complex showed a significant change in the residual interactions involved in the RAD50 and Mre11 docked complexes.

No mutant residue was found among the interacting residues in any docked complex, except for V117F, which was observed in the V117F–Mre11A complex. In the V117F–Mre11A docked complex, the V117F residue had hydrophilic interactions with the Mre11A peptide. This shows that 14 out of 15 residual variants are not involved directly in the docking. Results indicated that native C133F, Y1155D, R1156L, D1170H, M1197R, S1202, and G1226S docked complexes had same binding free energy ranges of 29.3–29.4 kcal/mol, whereas L518P, L1092R, N1144S, A1209P, and L1215P docked complexes had binding free energy ranges of −31.6 to −31.7 kcal/mol. A73P and V117F had binding free energies of −36.7–−35.9 kcal/mol. That showed more close interactions in the docked complexes than those in other complexes (Table 4). The seven docked complexes (L1215P, A1209T, N1144S, L1092R, L518P, V117F, and A73P) had slightly different to significantly different binding energies from native docked complexes. They were taken for the molecular dynamic simulation to observe the atomic and residual conformational alterations over time.

### 3.4 Molecular dynamic simulation

Molecular dynamic simulation revealed that while mutant models L518P, L1092R, N1144S, A1209T, and L1215P exhibited trends in conformational changes that were slightly different to the native model, mutants A73P and V117F showed significant alterations. Specifically, A73P and V117F had increased root mean square deviations and solvent-accessible surface area values, indicative of greater structural instability. These findings are consistent with those of previous studies, which suggest that any mutation in the critical functional domains of the RAD50 protein disrupts the interaction of RAD50 with Mre11A and conduct DNA repair (Deshpande et al., 2014; Bian et al., 2019).

**FIGURE 5:**
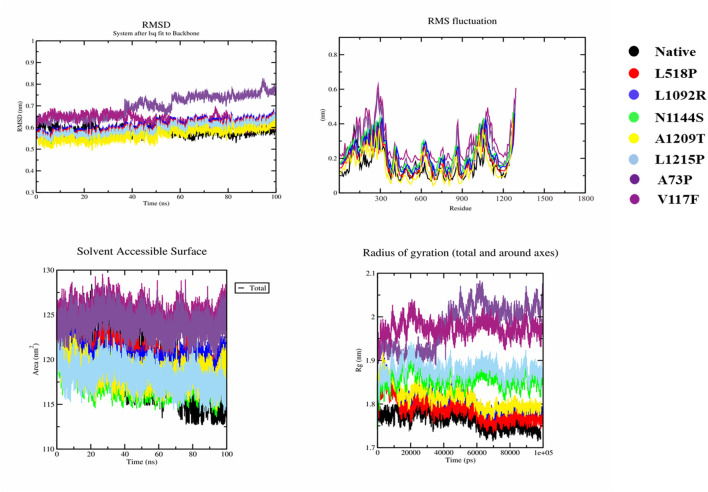
MD simulation results of native and seven mutant models. i) RMSD, root mean square deviation); ii) RMSF, root mean square fluctuation); iii) SASA, solvent accessible surface area, and iv) Rg, radius of gyration). Color schemes of all native and mutant models are provided, respectively.

However, all seven mutant models showed different behavior from the native model. Figure 5 depicts that the native model had an average RMSD value of 0.58 ns, with a range of 0.51–0.67 ns for all conformations over 100 ns. In contrast, L518P, L1092R, and L1215P had the same average RMSD value of 0.601 ns with a range of 0.56–0.64 ns, as did for N1144S. However, A1209T had an average RMSD value of 0.59 with a range of 0.495–0.59 ns. A73P and V117F, on the other hand, showed a significant divergence having an average RMSD of 0.73 ns and 0.64 ns with ranges 0.59 ns–0.82 ns and 0.49 ns–0.70 ns, respectively. Similar behavior was observed in RMSF, where the first five variants showed a little divergence from the native model, whereas A73P and V117F had a significant residual conformational alteration. SASA plots showed that the native model had decreasing values with time, with a range of 112.6–121 nm^2^, whereas N1144S and L1215P had the same range of 113–123 nm^2^. L518P, L1092R, and A1209T had SASA values ranging from 116 to 124 nm^2^, 117–123 nm^2^, and 115.5–123 nm^2^, respectively. A73P and V117F, on the other hand, showed the diverse range of 120–128.5 nm^2^ and 121–129.5 nm^2^, respectively. A similar behavior was observed in the radius of gyration where native models had the lowest gyration (1.72 nm–1.83 nm), and all the mutant models except A73P and V117F had a gyration range between 1.75 nm and 1.93 nm. A73P and V117F had a gyration range of 1.89 nm–2.08 nm and 1.90 nm–2.04 nm, respectively. MD simulation results revealed significant conformational alterations in the A73P and V117F mutant model, distinguishing them from all other mutant models.

## 4 Discussion

Deshpande et al. (2014) investigated several RAD50 mutants using forecasting mold implements and computer analysis of complete genome compositions and their effects on protein uprightness and binding properties (Deshpande et al., 2014). Our focus is on the infective effects of a subset of RAD50 mutants on Mre11A engagements. However, such extensive surveys provide a comprehensive overview of the RAD50 gene’s mutational context. To better explain RAD50’s contribution to homologous recombination and DNA damage response procedures, the current combined methodological system assembles a complete database of infective variation, (1999; Linh et al., 2017).

Our study revealed six significant nsSNPs in RAD50 (A73P, V117F, L518P, L1092R, N1144S, and A1209T) that bind to the protein and Mre11A during DNA double-strand break repair. Our analysis provides deeper insights into the broad conclusions of Kucukkal et al. (2015) and Linh et al. (2017) regarding the effect of the RAD50 mutant on the entire MRN complex. The prior exploration demonstrates the possibility of communicating by analyzing simultaneously the effect of the mutant on Mre1A itself, the entire repair involved, and the different DNA damage response components. The study sought to identify the precise RAD50 mutant to explain why the alteration of RAD50–Mre11A contact causes more and more diseases and curative development (Huang and Zhou, 2021).

This study revealed that the RAD50 mutant models A73P, V117F, L518P, L1092R, N1144S, and A1209T destabilize the protein structure, thus preventing the exchange of Mre11A within the DNA double-strand break repair MRN complex. The aforementioned RAD50–Mre11A functional break demonstrates that these mutant strains probably cause DNA repair channel impairments. These lead to the genomic uncertainty of the hallmark characteristic of cancer and familial hereditary disorders similar to the NBS (McCarthy et al., 2022; Bian et al., 2019). The A73P and V117F mutant proteins show maximum systematic deviations which simultaneously constitute excellent potential curative targets.

The secondary organization prediction combined with protein–protein docking simulation confirms the organizational change triggered by the RAD50 mutant. Despite the fact that many mutant types depart from the original structure, particularly in the cyclic and beta-sheet regions, secondary organization components show minimal changes. Protein–protein docking processes, as well as imitation research, reveal that mutant complexes exhibit distinct binding exchanges, particularly using hydrophilic–hydrophobic inclination when compared to the original, although a couple of mutants exhibit unique binding properties.

Docking simulation suggests that GLU75, Gln97, and Arg98 form adhesion contacts during A73P mutant compound formation. The disease-causing mutant shows those fresh residues that do not exist at the native binding site during the indication of protein movement. The study of the V117F–MRE11A complex revealed four residues (Lys71, Gln74, Ala73, and Glu75), which might have helped stabilize the arrangement and architectural activity due to the receptor properties of lysine. The experimental data show that residues located at a distance from the primary adhesion location are likely to induce to allosteric changes throughout the protein (Amor et al., 2016; Wu et al., 2022).

These communications, which are located far from the standard adhesion site, help stabilize the protein framework, thus ensuring correct conformational rigidity. This finding is consistent with those of the previous studies focused on the role of allosteric residues in the long-range effect on protein configuration and function (Tzeng and Kalodimos, 2012; Cui and Karplus, 2008). The protein maintains its functional conformation during mutational changes by maintaining the necessary motif and structure fold (Wu et al., 2022). To examine simultaneously allosteric phenomena and their effects on the protein structure and enzymatic durability, an investigative team used molecular movement simulations. A decrease in the space between the interactive components may disrupt the normal protein flexibility that the protein complex needs to perform its functions. The tighter binding of A73P and V117F may cause this interference with the proper flexibility of the RAD50 protein required for the MRN complex to perform its usual role in the impairment of DNA end resection and synapsis formation (Lafrance-Vanasse et al., 2015).

Molecular dynamics simulation results indicated a significant conformational alteration in the native and mutant RAD50 protein, particularly in the A73P and V117F mutants that showed increased RMSD and SASA values. These results suggested more structural instability in these mutants, which is consistent with the previous studies reporting that a mutation in the critical functional domain of the RAD50 protein disturbs its interaction with Mre11A, leading to the impairment of DNA repair mechanisms (Chansel-Da Cruz, Marie, et al., 2020). The unusual behavior of A73P and V117F may be attributed to their proximity to the NBD site. These mutations likely interfere with the conformational dynamics of this critical region. This may impair RAD50`s ability to properly interact with the MRE11 peptide and may disrupt the ATP-dependent DNA repair functions.

This study, which is consistent with previous studies, suggests that RAD50 mutations seem to underpin weakened DNA repair capacity and increased genome instability, which might play a pathophysiological role. For instance, the mutation of RAD50 has been associated with various diseases, including ataxia-telangiectasia, which is characterized by immaturity in DNA repair mechanisms and cancer-associated complications (Chisada et al., 2023). Although the comprehensive structural and functional analysis studies are likely to include some of these disease-related concepts, their focus is on more general treatment approaches. These studies propose that understanding the functional implications of these mutations enables therapeutic targeting, including small-molecule therapy or gene-editing strategies for diseases associated with RAD50 dysfunction (Yang et al., 2015). The present study focuses on specific therapies toward certain types of RAD50 mutants. However, the comprehensive analysis may consider more global treatment modalities, such as the patterning of the entire DSB repair pathway or designing small molecules that stabilize or correct the association between RAD50 and Mre11A.

These studies employ computational predictions of the consequences of mutations and experimental validation of the results. However, the studies’ focus is different. Based on the defined mutants, the current study examines the functional outcomes of each one in more depth, for example, protein stability analysis and co-immunoprecipitation to confirm disruptions (Rentzsch et al., 2019). On the other hand, it is possible that the comprehensive structural and functional analysis uses more extensive computational simulations to analyze the structural consequences of multiple mutations. It mirrors high-throughput strategies to estimate how these changes affect RAD50’s stability and functionality in different cellular environments (Capriotti and Fariselli, 2017).

Though existing studies have broadly demonstrated that mutations in RAD50 are substantially involved in DNA repair and genomic stability, this study provides a targeted focus on a specific subset of pathogenic nsSNPs, offering a comprehensive insight into how these mutations impair RAD50–Mre11A interactions. By combining computational tools with structural analysis, this study advances our understanding of RAD50’s role in the DNA repair pathway. In contrast, comprehensive structural and functional analyses have historically taken a more generalized approach, focusing on the broader implications of RAD50 mutations across the MRN complex. Together, these viewpoints offer complementary insights to help us better comprehend certain mutations for the development of targeted therapies.

## 5 Conclusion

This study highlights the pathogenic impact of specific RAD50 nsSNPs (A73P, V117F, L518P, L1092R, N1144S, and A1209T) on its interaction with Mre11A, emphasizing their critical roles in DNA double-strand break repair. Among these, A73P and V117F mutants demonstrated the most significant structural alterations, with increased RMSD and SASA values, indicative of greater instability. These mutations, located near the nucleotide-binding domain, potentially interfere with RAD50’s conformational dynamics, disrupting ATP-dependent DNA repair functions. The study underscores the role of allosteric interactions in mediating protein flexibility and stability, which is consistent with prior findings on RAD50’s involvement in genomic stability and disease pathways. These insights pave the way for targeted therapeutic approaches, such as small-molecule stabilization or gene editing, to mitigate the impact of RAD50 mutations on DNA repair mechanisms.

The 100 ns simulation length poses a limitation, which may not fully capture long-term protein dynamics, especially for a large protein like RAD50. Although computational approaches provided insights, experimental validation through mutagenesis or binding assays is necessary. Broader analyses including more RAD50 mutations and their interactions with the entire MRN complex are required for a comprehensive understanding. Advanced simulation techniques, integration of machine learning, and *in vivo* studies could further improve the interpretation of RAD50’s structural and functional impacts.

## Data Availability

The raw data supporting the conclusions of this article will be made available by the authors, without undue reservation.

## Abbreviations

nsSNPs, non-synonymous single-nucleotide polymorphisms; MRN, Mre11–Rad50–Nbs1; DSBs; DNA, double-strand breaks; HR, homologous recombination; NHEJ, non-homologous end joining; hRAD50, RAD50 gene in humans; NBS, Nijmegen breakage syndrome; SNPs, single-nucleotide polymorphisms.
